# Injectable temperature-sensitive hydrogel facilitating endoscopic submucosal dissection

**DOI:** 10.3389/fbioe.2024.1395731

**Published:** 2024-04-29

**Authors:** Ruifen Xu, Xiaoyu Yang, Tong Yi, Tao Tan, Zhongqi Li, Xuyang Feng, Jing Rao, Pinghong Zhou, Hao Hu, Yonghua Zhan

**Affiliations:** ^1^ Anesthesiology Department, Shaanxi Provincial People’s Hospital, Xi’an, Shaanxi, China; ^2^ Engineering Research Center of Molecular and Neuro Imaging of the Ministry of Education, School of Life Science and Technology, Xidian University, Xi’an, Shaanxi, China; ^3^ University of Shanghai for Science and Technology, Shanghai, China; ^4^ Endoscopy Center and Endoscopy Research Institute, Shanghai Collaborative Innovation Center of Endoscopy, Zhongshan Hospital, Fudan University, Shanghai, China; ^5^ Department of Cardiology, Xijing Hospital, The Fourth Military Medical University, Xi’an, Shaanxi, China

**Keywords:** endoscopic mucosal dissection, submucosal injection, mucosal elevation, gastrointestinal tumors, hydrogel

## Abstract

**Purpose:** Early gastrointestinal tumors can be removed by endoscopic procedures. Endoscopic mucosal dissection (ESD) requires submucosal fluid injection to provide mucosal elevation and prevent intraoperative perforation. However, the clinically applied normal saline mucosal elevation height is low for a short time, which often requires multiple intraoperative injections that increase the inconvenience and procedure time. In addition, recently researched submucosal injection materials (SIM) suffer from complex preparation, poor economy, and poor biocompatibility. Therefore, there is an urgent need for a new type of SIM that can provide long, safe and effective mucosal elevation in support of the endoscopic procedures.

**Methods:** The FS hydrogel is based on polyethylene-polypropylene glycol (F-127) mixed with sodium alginate (SA). The different physicochemical properties of FS hydrogels were characterized through various experiments. Afterward, various biosafety assessments were carried out. Finally, the performance of FS hydrogels was evaluated by *in vitro* submucosal injection and *in vivo* swine ESD.

**Results:** The experimental results show that the FS hydrogel is liquid at room temperature, making it easy to inject, and when injected under the mucosa, it undergoes temperature-induced cross-linking, transforming from a liquid to a solid state to provide long-lasting mucosal augmentation. At the same time, the FS hydrogel exhibits controllable gelation, stability, and biocompatibility. The results of *in vitro* submucosal injections and *in vivo* ESD procedures showed that FS achieves high mucosal augmentation and provides good submucosal cushioning in the long term.

**Conclusion:** In summary, the F-127/SA hydrogel is simple to synthesize, cost-effective, safe, easy to store, and able to assist ESD well from the perspective of practical clinical problems, indicating that the FS hydrogel can be an ideal potent submucosal injection substitution.

## 1 Introduction

According to the statistics, the incidence and mortality of gastrointestinal cancers remain high, posing a serious threat to human health (Sung et al., 2021). The gastrointestinal (GI) tract wall can be divided into four layers: mucosa, submucosa, muscularis propria, and serosa (Hussey et al., 2018). Early GI tumors are lesions that infiltrate only within the submucosal layer and can be removed endoscopically (Nie et al., 2019). Endoscopic submucosal dissection (ESD) has been clinically proven to be an excellent treatment for early GI tumors. However, it is associated with the risk of causing perforation of the GI tract and bleeding during endoscopic resection (Takamaru et al., 2016; Du et al., 2022; Pimentel-Nunes et al., 2022). Submucosal injection materials (SIM) can elevate the mucosal lesion by forming a fluid cushion between the mucosa and intrinsic muscular layer, providing sufficient operating space for mucosal resection and avoiding the electrical and thermal damage that may be caused during endoscopic resection (Yeh and Triadafilopoulos, 2005; Ferreira et al., 2016).

Normal saline has become the clinic’s most commonly used submucosal injection material due to its biocompatibility, wide availability, low cost, and low toxicity (Takahashi et al., 2009). However, it is readily absorbed by the surrounding tissues and cannot provide prolonged mucosal elevation. Normal saline often needs to be injected multiple times to resect larger lesions. This significantly increases the inconvenience of the procedure, prolongs the duration of the procedure, and may result in complications, such as intraoperative hemorrhage caused by the injections. Therefore, there is an urgent need for a new type of SIM that can provide prolonged and effective mucosal elevation in support of ESD procedures.

Several solutions have recently been used to achieve prolonged elevation of SIM, including hypertonic, high-viscosity, and hydrogels. However, as research progresses, most SIMs face the challenge of not being able to combine low injection pressure with long-lasting elevation capability. For example, hypertonic solutions (hypertonic saline, dextrose, glycerol, hydroxypropyl methylcellulose, etc. (Yandrapu et al., 2017; Wang et al., 2021) provide low injection pressure, but their ability to provide sustained elevation has not been demonstrated to be superior to that of normal saline (Fujishiro et al., 2004). High-viscosity solutions could theoretically provide better elevation, but high viscosity means difficult injections. Then researchers experimented with low-concentration solutions, such as 0.4% hyaluronic acid, which is approved for clinical submucosal injection in Japan. Hyaluronic acid has been shown in several studies to provide longer-lasting submucosal augmentation, higher ESD resection rates, and lower complication rates (Yamamoto et al., 2002; Kim et al., 2015), but it is less economical and may stimulate the proliferation of residual tumor cells (Matsui et al., 2004).

Meanwhile, injectable hydrogels have been widely used in tumor resection, drug delivery and wound treatment, and their biosafety has been widely recognized (Feng et al., 2023; Jin et al., 2023; Kang et al., 2023). To address the dilemma faced by SIM, researchers have turned their attention to hydrogels that can be cured *in situ* (Dumortier et al., 2006; Nguyen et al., 2016; Kim et al., 2017; Cao et al., 2019; Kim et al., 2021). Various hydrogel solutions have been proposed, for example, a chitosan-based photocrosslinked hydrogel designed by Masayuki et al. Experimental results showed a significant improvement in the strength and duration of mucosal augmentation (Kumano et al., 2012). However, this requires the use of an ultraviolet light source during the procedure, which adds inconvenience to the endoscopic procedure and is difficult to replicate, not to mention it may cause inflammation and carcinogenic effects. Ryohei et al. researchers chose to use a two-component hydrogel (calcium chloride and sodium alginate), in which a solution of calcium chloride is injected into the submucosa, followed by the injection of a portion of sodium alginate into the initial injection site (Hirose et al., 2021). This method provides long-lasting, high-intensity elevation, but it requires multiple injections and may require waiting for the calcium chloride solution to be absorbed, significantly increasing the time required for the procedure. A small number of researchers have also begun to focus on the performance of shear-thinning materials for SIM (Pang et al., 2019). Yan Pang et al. have developed a shear-thinning hydrogel with low injection pressure and good elevation performance (Tang et al., 2022), but the biocompatibility and maneuverability of shear-thinning materials are unknown.

**SCHEME 1:**
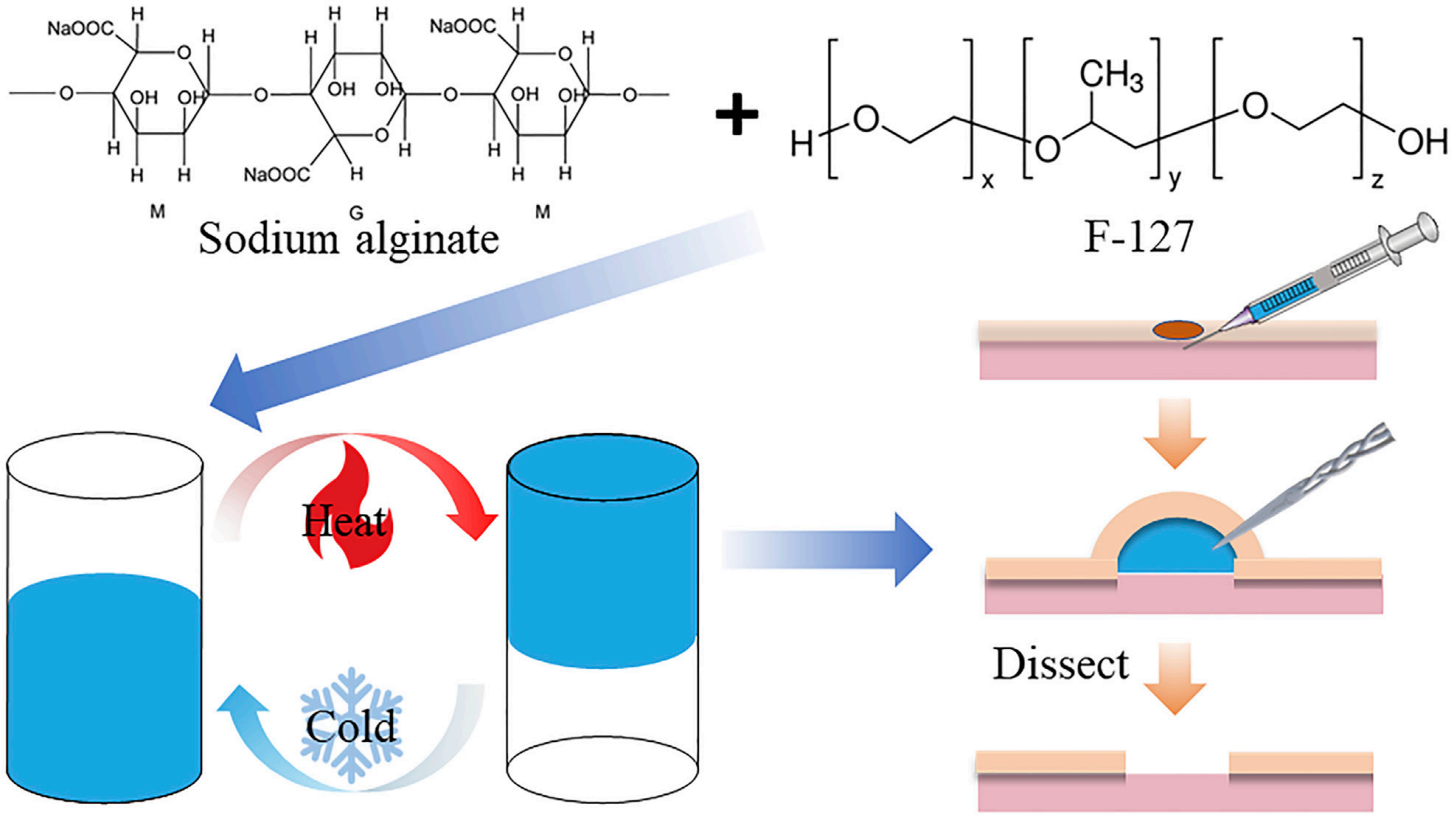
Schematic of FS hydrogel-assisted ESD.

Therefore, how to strike a balance between easy injection and long-lasting elevation, with consideration of cost-effectiveness, remains a major challenge in SIM development. In this article, our research ambition is temperature-sensitive hydrogels. In the past, the synthesis method for these hydrogels was complicated and the materials used were expensive. To address these challenges, we have successfully developed a temperature-sensitive hydrogel (FS), which is readily available, inexpensive, easy to prepare, has good biocompatibility, is easy to inject, and is capable of long-lasting mucosal elevation (Scheme 1). *In vivo* and *in vitro* results showed that FS could achieve high mucosal augmentation, and provide good submucosal cushioning over a long time, which indicates that the FS hydrogel can be an ideal SIM for endoscopic procedures.

## 2 Materials and methods

### 2.1 Materials

Pluronic F127, PBS phosphate buffer, L-glutamine (L-G) and 2-[4-(2-hydroxyethyl) piperazin-1-yl] ethane sulfonic acid (HEPES) were purchased from Solarbio. Sodium alginate (SA) and Dimethyl sulfoxide (DMSO) were purchased from Aladdin. Methylene blue was purchased from Maclean’s. Fetal bovine serum (FBS) was purchased from Gibco. Culture medium (DMEM) and Trypsin were purchased from Hyclone. Double antibody (A/P) was purchased from Biosharp. Counting Kit-8 (CCK-8) was purchased from Dojindo. Paraformaldehyde fixative (4%) was purchased from Beyotime. An immune factor test kit was purchased from Servicebio. Isoflurane was purchased from Shandong Ante Herding Technology Co.

### 2.2 Preparation and characterization of FS hydrogels

The temperature-sensitive hydrogel material was prepared by the low-temperature method. About 0.95 g of Porosam 407 (F-127) and 0.005 g of sodium alginate (SA) were added into 5 mL of ultrapure water along with 75 μg of methylene blue. These were then placed on a shaking table at 200 rpm for 10 min, and into a 4 °C refrigerator for 12 h for low-temperature solubilization to obtain the temperature-sensitive hydrogel; then they were put in 4 °C for preservation away from light. The temperature-sensitive hydrogel was preserved at 4 °C and protected from light. Afterward, the FS hydrogels were characterized by using a Hitachi SU7000 scanning electron microscope (SEM) to analyze the microscopic morphology of the materials, a TA HR20 rheometer was used to evaluate the rheological properties of the FS hydrogels, and an RVA-TM rapid viscosity analyzer was utilized to determine the rotational viscosity. FS hydrogels were placed in a water bath at 37°C and the time required for gelation was evaluated based on the change in conductivity. Then, the swelling properties, degradation rate, and stability of the hydrogels were evaluated by conventional experiments.

### 2.3 Biocompatibility evaluation of FS hydrogels

The experimental protocols were approved by the Animal Care and Use Committee of Zhongshan Hospital, Fudan University (2017-040). Cytotoxicity of FS hydrogels was verified using Counting Kit-8. After that, the rats were anesthetized with gas, 200 μL of FS hydrogel was injected subcutaneously on the back and 50 μL of FS hydrogel was injected intramuscularly in the legs of rats in the experimental group. Rats were anesthetized by gas anesthesia at a dose of 0.41 mL/min at 4 L/min fresh gas flow (2% isoflurane) for 2–3 min. Control rats were injected with the same dose of normal saline at the same location as negative controls, and cell morphology of the skin and muscle at the injection site was observed by Hematoxylin Eosin staining (H&E staining) to determine the damage to the tissues. Rats injected with FS hydrogel subcutaneously were divided into two blood samples, where one blood sample was tested for blood biochemical indices and the results were analyzed. The second blood sample was used to determine the levels of Interleukin-1β (IL-1β), Interleukin-6 (IL6), and tumor necrosis factor-α (TNF-α) by enzyme-linked immunosorbent assay (ELISA) to assess whether the FS hydrogel induces an inflammatory response *in vivo*. Hemolytic toxicity of the FS hydrogel was determined using sheep erythrocytes.

### 2.4 *In vitro* injection and mucosal elevation of the FS hydrogel

*In vitro* injection experiments were conducted to observe the elevation properties of the FS hydrogel. Since the upper 1/3 of the porcine stomach was similar to the thickness and histological structure of the human stomach, the upper 1/3 of the porcine stomach was selected as the injection site for the *in vitro* simulated human ESD experiments (Zhu et al., 2019). Fresh porcine stomachs purchased from the market were used for this purpose, and normal saline with an equal concentration of methylene blue was used as the negative control. The porcine stomach was cut into regular 5*5 cm shapes and immersed in a water bath at 37 °C. Using a 5 mL syringe connected with a 23G4 disposable endoscopic needle (Boston Scientific), 2.5 mL of temperature-sensitive hydrogel was injected into the submucosa, and a normal saline group was set up as the control. Photographs were taken and recorded 1 min, 3 min, 5 min, 10 min, 20 min, 30 min, 60 min, 90 min, and 120 min before and after injection, and the height of elevation of the injection site was measured with a vernier caliper, respectively. The height of the injection site was measured with a vernier caliper, and the whole process was carried out in a 37 °C water bath before and after the injection, except for taking pictures and measuring the height, to ensure that the environment of the *in vivo* digestive tract was simulated as much as possible.

### 2.5 *In vivo* ESD model of the FS hydrogel

The *in vivo* ESD procedures were performed in experimental pigs with the assistance of Zhongshan Hospital of Fudan University. The experimental pigs were fasted for 24 h before the experiment, and after general anesthesia with tracheal intubation, endoscopy was used to locate the simulated esophageal tumor. 2 × 2 cm of mucosa was selected on the esophagus to simulate the tumor site, and then FS hydrogel was injected into the submucosal layer. The amount of hydrogel injected was judged according to the operation until the mucosa of the simulated lesion was sufficiently elevated, and then ESD excision was performed. The entire procedure was videotaped, and the endoscopist filled out a surgical evaluation form to assess the differences between the normal saline group and the FS hydrogel group in terms of surgical time, injection volume, resection efficiency, intraoperative muscle injury, and intraoperative perforation. The resected mucosa in the ESD experiment was stained by H&E and subjected to histological observation.

## 3 Results and discussion

### 3.1 Synthesis and characterization of the FS hydrogel

**FIGURE 1:**
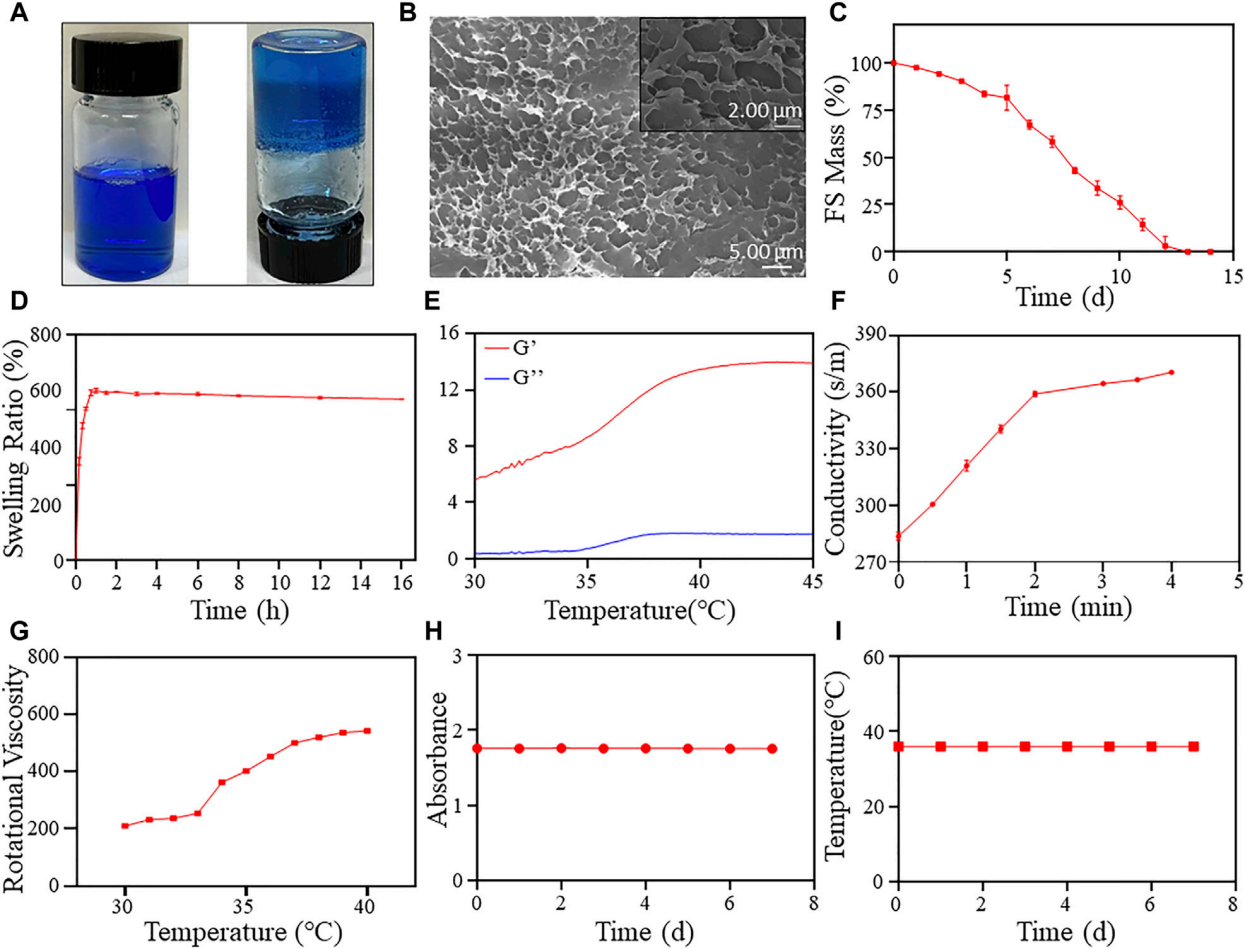
FS hydrogel characterization. **(A)** Hydrogel appearance (left at room temperature, right at body temperature); **(B)** SEM image of the FS hydrogel; **(C)** Degradation rate of the FS hydrogel in PBS solution; **(D)** The swelling rate of the FS hydrogel in PBS solution; Evaluation of rheological properties of **(E,F)** the FS hydrogel. **(E)** Storage modulus and loss modulus; **(F)** Changes in conductivity; **(G)** Rotational viscosity of the FS hydrogel at 30°C–40 °C; **(H)** Stability of the FS hydrogel; **(I)** The change of phase transition temperature over time.

Developing a temperature-sensitive hydrogel material for clinical ESD submucosal injection is crucial. The hydrogel should be safe, cost-effective, easy to synthesize, and exhibit excellent performance. The detailed synthesis process is shown in Scheme 1. After dissolving F-127 and SA in ultrapure water, the hydrogel turned blue and transparent due to the addition of methylene blue dye, as seen in Figure 1A. The hydrogel’s color makes it an ideal indicator for the injection site. Additionally, the material transitions from a liquid to a solid state upon heating, demonstrating that the FS hydrogel could achieve both low injection pressure and long elevation performance by SIM. To formulate the hydrogel, several experiments were conducted to screen for appropriate formulations (Supplementary Table S1). Further studies were conducted to characterize the FS hydrogel. The SEM results indicate that F-127 and SA dissolved in water and self-crosslinked to form a uniform three-dimensional network structure, confirming the successful synthesis of the hydrogel (Figure 1B). Subsequently, to ensure biodegradability of the FS hydrogel in the human body, it was placed in a PBS solution at 37°C for observation. The degradation rate increased gradually with time, and the hydrogel was completely degraded after 12–13 days (Figure 1C). Given the complex environment of the gastrointestinal tract and stomach, it is expected that the FS hydrogel will degrade more rapidly *in vivo*. This characteristic accelerates the process of cleaning and healing clinical postoperative wounds.

Furthermore, the FS hydrogels demonstrated favorable swelling properties in PBS solution (Figure 1D). The swelling rate of the FS hydrogels increased rapidly during the first 45 min and reached equilibrium after that. The swelling rate of the FS hydrogel decreased with increasing soaking time in PBS. The advantageous swelling characteristics of the FS hydrogel may protect the wound during clinical mucosal debridement of ESD. To further characterize the physicochemical properties of FS hydrogels, we determined their rheological properties and rotational viscosity (Figures 1E–G). The energy storage modulus (G′) of the hydrogel increased from 5.66 Pa to 13.83 Pa between 30°C and 45 °C, while the elastic modulus (G″) increased from 0.37 Pa to 1.81 Pa (Figure 1E). G′ is always higher than G″ throughout the process, and the difference between the two increases with increasing temperature. This indicates that the viscosity of the FS hydrogel is increasing and its state is changing from liquid to solid. After that, we calculated the loss factor of FS using G′ and G'' (Supplementary Figure S1). The point at which the loss factor transitions from rising to falling typically corresponds to the temperature at which the hydrogel undergoes a liquid-solid transition. In this case, the inflection point was calculated to occur at 37.82°C. This is because the actual solidification temperature is delayed due to the rheometer setting’s slightly faster temperature rise and the test material’s slower thermal conductivity. The hydrogel’s liquid-solid transition temperature should be in the range of 36°C–37°C, which corresponds to the final endoscopic solidification temperature. The FS hydrogel meets the requirement of forming a solid gel in the submucosal layer. Subsequently, we conducted experiments on the gelation time required for FS hydrogels. The findings show that the conductivity in FS hydrogels reaches its maximum value at about 3 min, and by this time the internal temperature has reached 37°C. This suggests that FS hydrogels can achieve rapid gelation at 37°C, which contributes to better mucosal elevation. The rotational viscosity measurement results indicate an increase during the warming process at 30°C–40°C, followed by a sudden increase between 34°C and 37°C (Figure 1G). In the clinical environment of ESD, the FS hydrogel is in a low-viscosity injectable state at room temperature. However, upon injection into the submucosal layer, the viscosity of the FS hydrogel rapidly increases due to the body temperature environment, transforming it from a liquid to a solid state. This provides a long-lasting elevation effect.

The stability of the FS hydrogel was assessed by measuring its transmittance and phase transition temperature (Figures 1H, I). The results indicate that the absorbance of the hydrogel at 665 nm, where the absorption of methylene blue peaks, did not change significantly over 7 days (Figure 1H). The FS hydrogel remained clear and transparent throughout storage, and no flocculent precipitation was observed. The 7-day phase transition temperature test results indicate that the FS hydrogel consistently maintained a phase transition temperature of 36°C (Figure 1I). The hydrogel exhibits good stability and the ability to undergo continuous reversible phase transition, making it an ideal candidate for clinical ESD.

### 3.2 Cytotoxicity and hemolytic toxicity of the FS hydrogel

**FIGURE 2:**
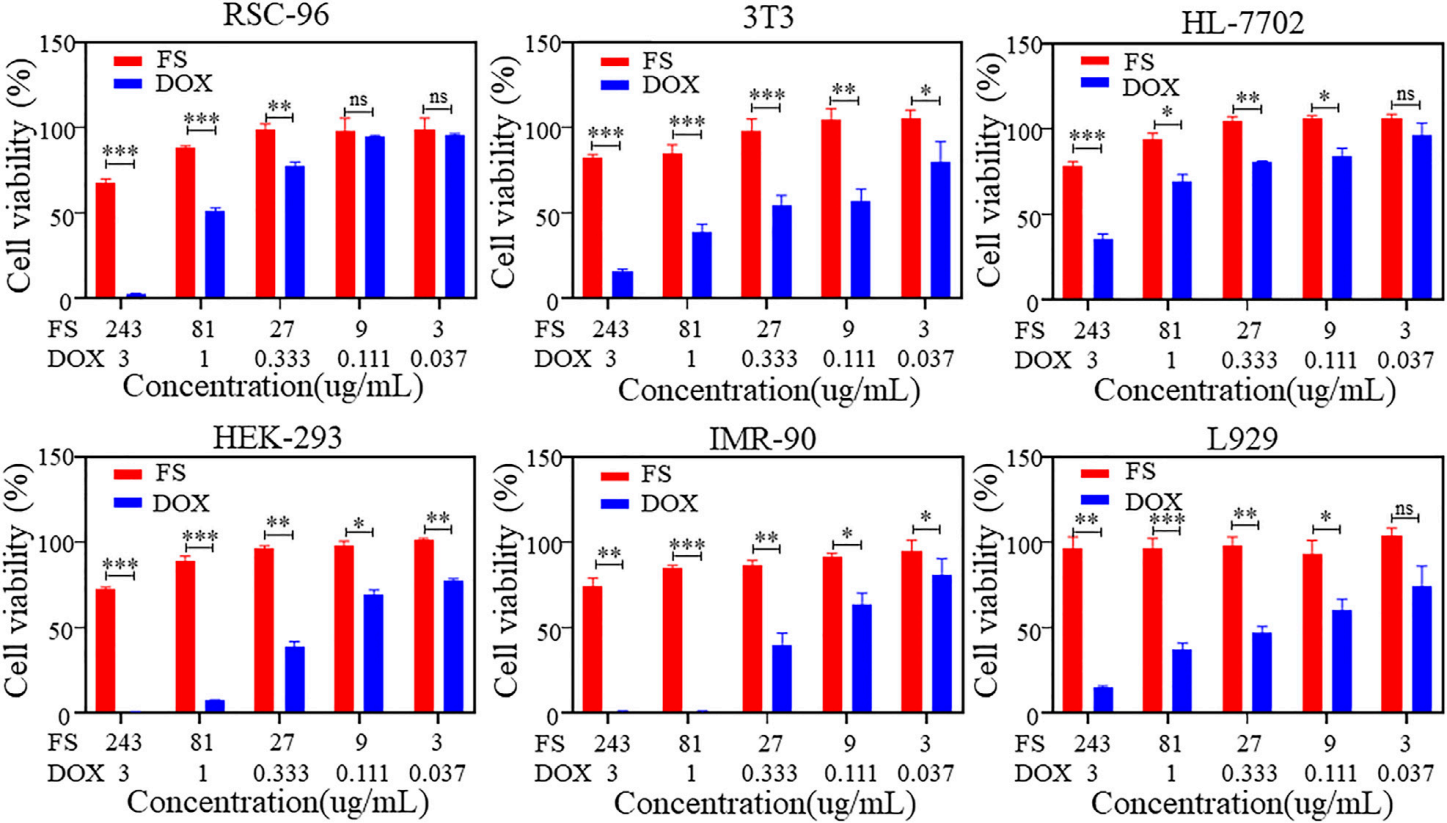
*In vitro* cytotoxicity of the FS hydrogels (iterate three times).

The biocompatibility of SIM is a crucial consideration in biomedical applications. To assess the cytotoxicity of FS hydrogels, we analyzed the cell viability of various cell types using the CCK-8 cell proliferation assay. Figure 2 displays the results of the cytotoxicity assay for rat Schwann cells (RSC-96), mouse embryonic fibroblasts (3T3), human normal hepatocytes (HL-7702), human embryonic kidney cells (HEK-293), embryonic lung fibroblasts (IMR-90), and mouse epithelioid fibroblasts (L929). Under the condition of gradually increasing DOX concentration, the cell viability of all three groups, RSC-96, HEK-293 and IMR-90, gradually decreased to 0, while the cell viability of the other three groups decreased to less than 50%, indicating significant cytotoxicity. The cell viability of the three groups of temperature-sensitive hydrogels also slightly decreased. However, increasing the concentration of the hydrogel material to 243 μg/mL resulted in a cell survival rate of over 50% for each group. This indicates that the temperature-sensitive hydrogel has low cytotoxicity and maintains relatively high biosafety performance even at higher concentrations.

**FIGURE 3:**
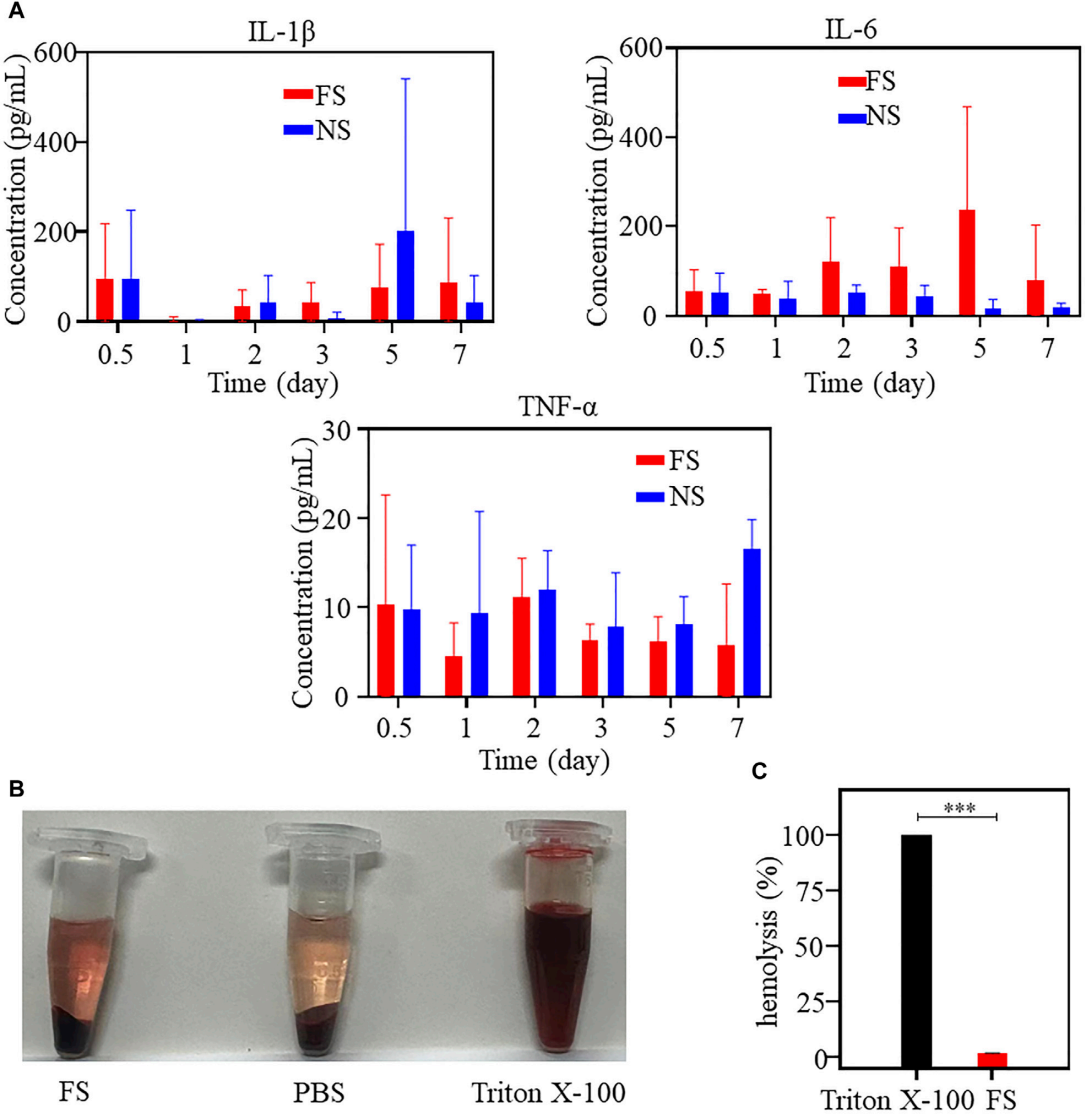
*In vivo* biocompatibility of the FS hydrogels (iterate three times). **(A)** Determination of immune factors of the FS hydrogel in an animal toxicology experiment; **(B,C)** Hemolysis experiment: **(B)** Hemolysis images of different samples; **(C)** Hemolysis rate of the positive control group and experimental group (iterate three times).

Furthermore, the results of *in vitro* hemolysis experiments using FS hydrogel indicate that the supernatant of the FS hydrogel group was slightly darker red, suggesting the presence of weak hemolysis (Figure 3B). The calculated hemolysis rate of FS hydrogel was 1.72% (Figure 3C), which was lower than that of drug treatment. These findings suggest that FS hydrogel is biologically safe and suitable for use in subsequent *in vivo* ESD procedures.

### 3.3 Animal toxicology of the FS hydrogel

**FIGURE 4:**
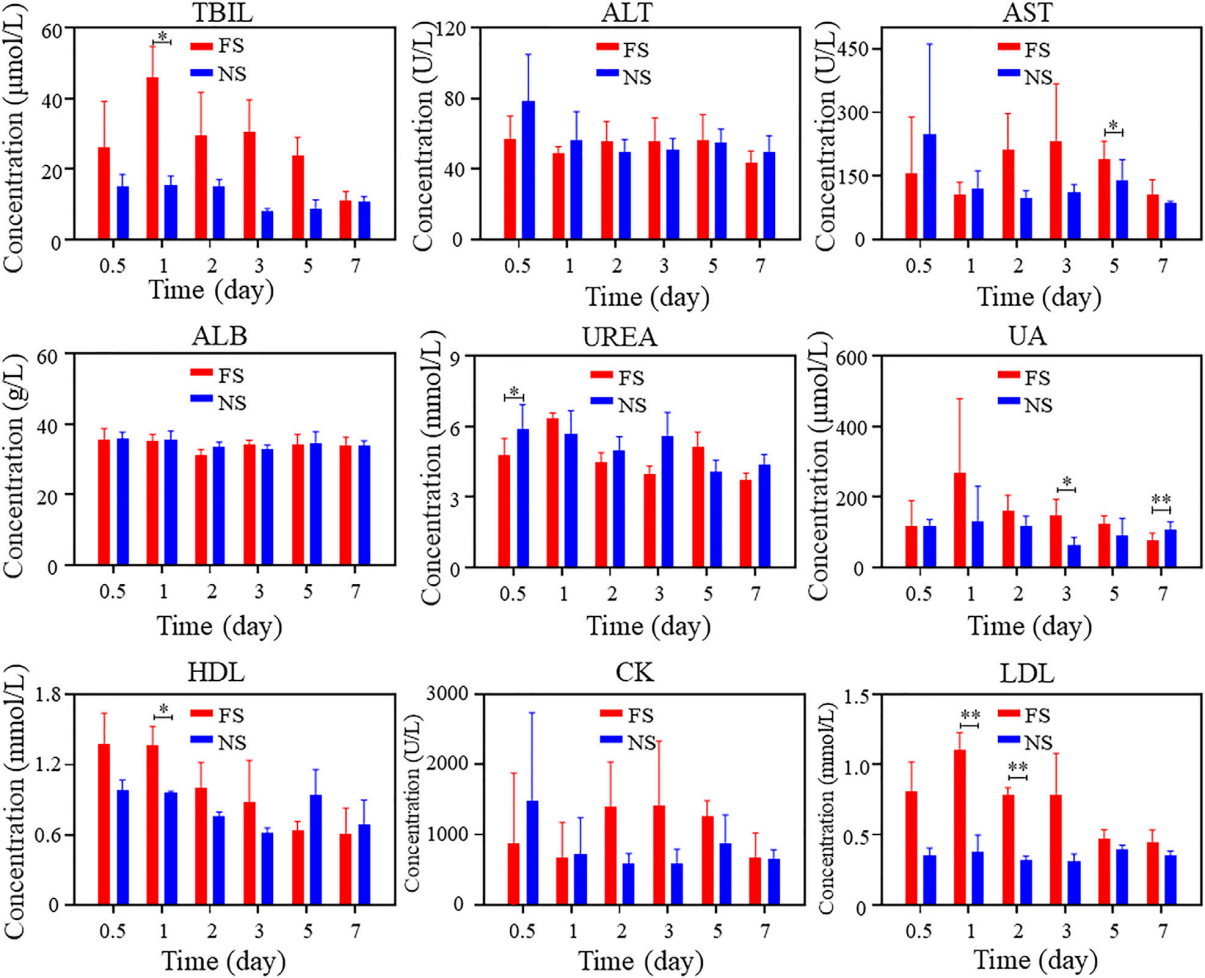
Blood biochemistry of FS hydrogels after subcutaneous injection (iterate three times).

To verify the biosafety of FS hydrogels *in vivo*, we conducted animal toxicology evaluations. Figure 3A displays the changes in immune factors after FS hydrogel injection in rats. No significant changes were observed in TNF-α, IL-6, and IL-1β levels in the FS hydrogel group compared to the normal saline group within 7 days after injection. This suggests that the injection of FS hydrogel did not cause a serious immune response in the body within 7 days. Meanwhile, the blood biochemical analysis revealed no significant differences in the counts of alanine aminotransferase (ALT), albumin (ALB), and urea (UREA). The total bilirubin (TBIL) count was elevated during the first 5 days and returned to normal levels by the seventh day. Furthermore, during the first few days, aspartate aminotransferase (AST), uric acid (UA), high-density lipoprotein cholesterol (HDL), creatine kinase (CK), low-density lipoprotein cholesterol (LDL), and other markers showed a slight elevation (Figure 4). This indicates that the FS hydrogel is biologically safe *in vivo* and can be safely used in future studies.

**FIGURE 5:**
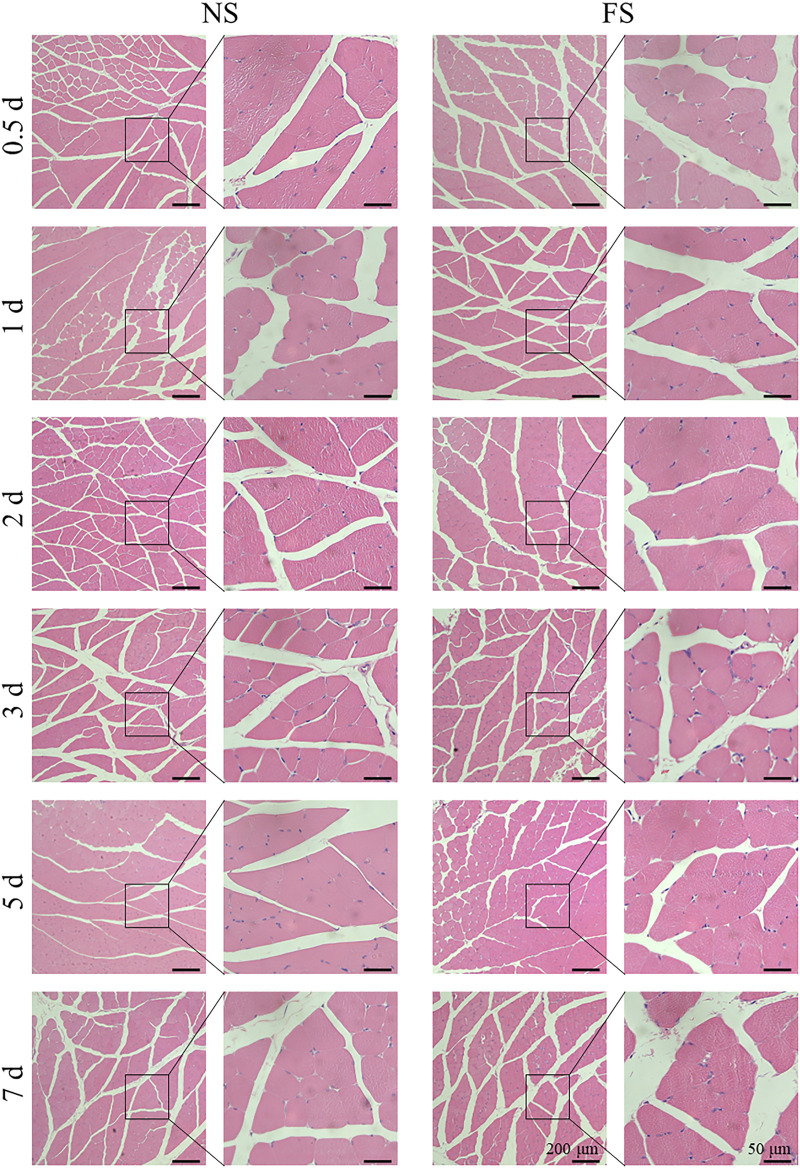
Damage to muscle tissue by the FS hydrogels. NS, normal saline.

**FIGURE 6:**
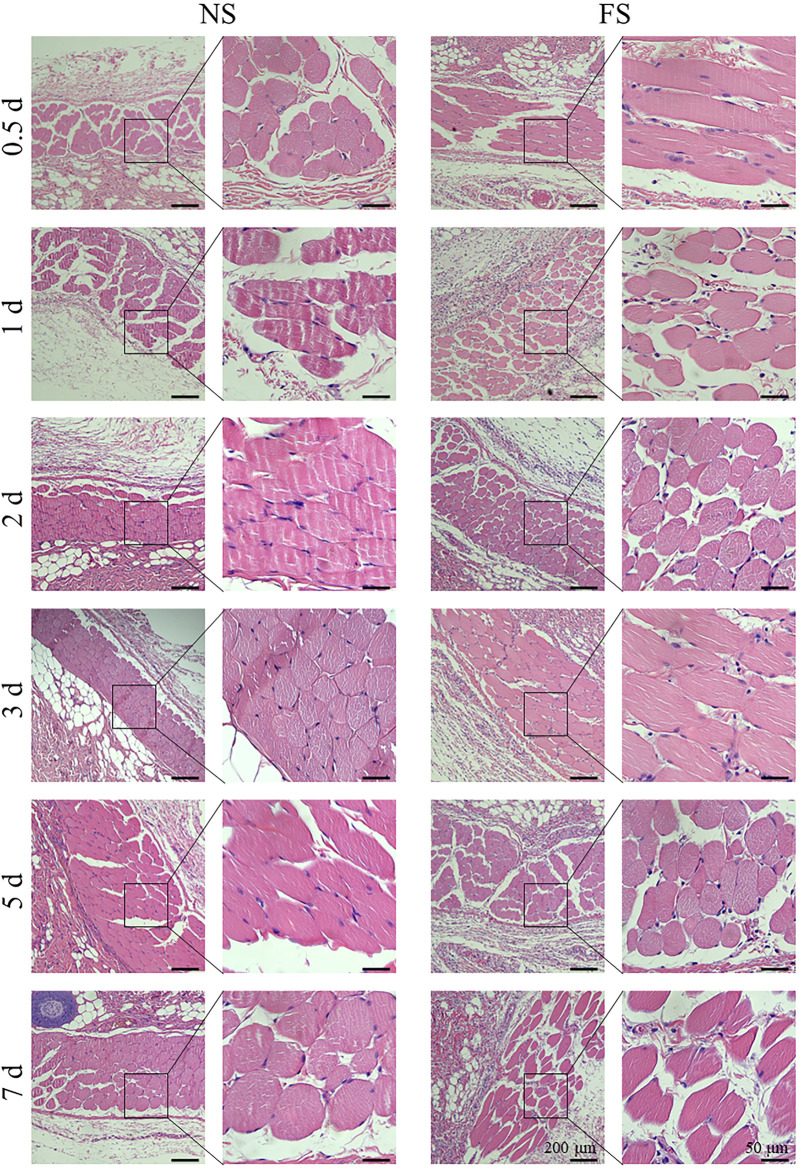
Damage to skin tissue by the FS hydrogel.

To clarify the effect of the FS hydrogel on tissues, we used H&E staining for histological analysis of the muscle injection site and skin injection site of SD rats in each group. Subcutaneously, 200 μL was injected, and into the muscle, 50 μL was injected (Figure 5; Figure 6). Both the experimental group, injected with the FS hydrogel, and the control group, injected with normal saline, maintained uniform cellular morphology and complete cellular structure in both muscle and skin tissues at different times. This indicates that the FS hydrogel did not cause any noticeable tissue damage during the 7 days of intramuscular and subcutaneous injections. When used as an ESD submucosal injection, FS hydrogel can help maintain the integrity of the lesion, making it easier to assess the pathological staging later.

### 3.4 *In vitro* mucosal evaluation of FS hydrogels

**FIGURE 7:**
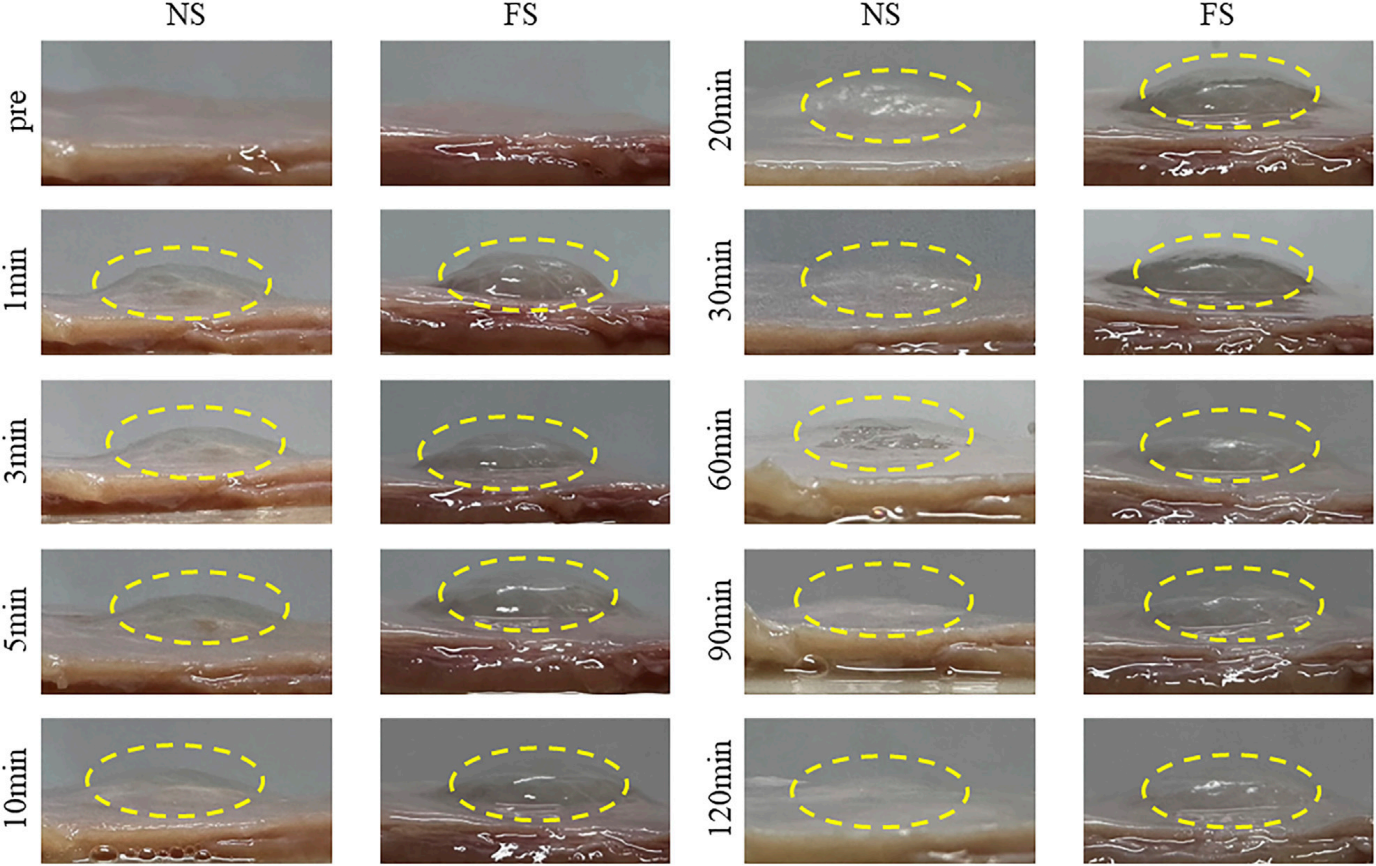
*In vitro* mucosal elevation capacity of the FS hydrogel.

**FIGURE 8:**
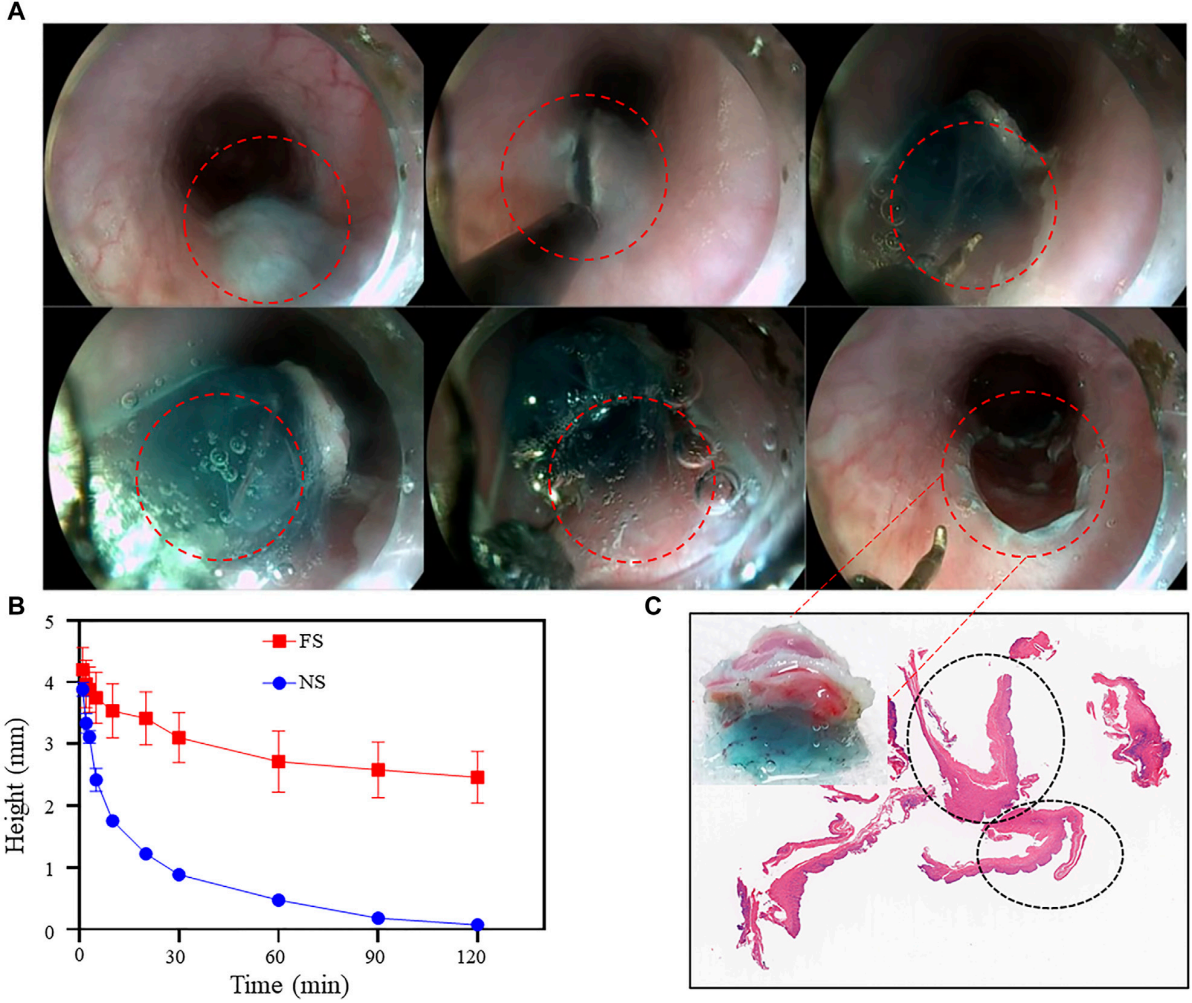
FS Hydrogel-assisted ESD. **(A)** Endoscopic images of the target site during and after submucosal injection of ESD; **(B)** Changes in protrusion height after simulated injection into the pig stomach *in vitro*; **(C)** Photo and section of mucosal tissue after ESD resection.

To assess the suitability of FS hydrogel for clinical ESD procedures, we utilized porcine stomach as an *in vitro* model. FS hydrogel was injected beneath the mucosa of the upper third of the porcine stomach, and the height of the elevation was monitored at different time points (Figure 7). In the normal saline group, the elevation height decreased rapidly within 30 min and then slowly between 30 and 120 min. In contrast, the bulge in the FS group was maintained at a constant level for 120 min. Additionally, the experiment measured the change in the height of mucosal elevation, which decreased from 4 mm to approximately 1 mm within 30 min in the normal saline group. However, in the FS group, the height only decreased to 3 mm (Figure 8B). The experimental results suggest that the FS hydrogel has a longer elevation time and intensity after submucosal injection compared to normal saline. Therefore, the FS hydrogel can be used for subsequent *in vivo* animal experiments.

### 3.5 *In vivo* FS hydrogel assisted ESD

After ensuring the biocompatibility and submucosal elevation properties of the FS hydrogel, ESD procedures were performed on the experimental pigs. Endoscopic images showed that the FS hydrogel formed a gel *in situ* after injection into the submucosa and provided good support throughout the procedure (Figure 8A). Feedback from the endoscopists indicated that FS hydrogel was slightly less difficult to perform than normal saline (Supplementary Videos S1, S2).

Two cases of intra-operative muscle injury were observed in the normal saline group, which is one of the common intra-operative risks in clinical ESD, mainly due to the insufficient strength of the elevation provided by normal saline. In contrast, no intramuscular layer injury was observed in the FS hydrogel group. The clinician’s intra-operative assessment form showed that the initial injection volume was similar in the normal saline and FS groups and no perforation occurred in either group. However, the normal saline and FS groups showed a significant difference in other assessment measures: the mean operative time in the normal saline group was 927 s, the mean total injection volume was 13.6 mL, and all samples required repeat injections during the procedure. In contrast, the FS group had a mean total operative time of 797 s, a mean total injection volume of 5.58 mL, and only two cases required repeat injections during the procedure (Table 1). This suggests that the use of FS hydrogel for endoscopic resection has a favorable safety profile and reduces the risk of complications compared to normal saline.

**TABLE 1:** ESD surgical evaluation.

Evaluation indicators	Normal saline (n = 8)	FS hydrogel (n = 8)	*p*-value
First volume of injection (mean ± SD, mL)	6.24 ± 0.77	5.09 ± 0.87	0.079
Total volume of injection (mean ± SD, mL)	13.60 ± 0.97	5.58 ± 0.76	<0.001
Total procedure time (mean ± SD, s)	927.50 ± 120.82	797.88 ± 51.62	0.031
Re-injection required (n, %)	8 (100%)	2 (25%)	0.007
Lesion size (mean ± SD, cm^2^)	4.19 ± 1.06	3.99 ± 0.84	0.49
*En-bloc* resection (n, %)	8 (100%)	8 (100%)	1
Intraoperative muscle injury (n, %)	2 (25%)	0 (0%)	0.47
Intraoperative perforation (n,%)	0 (0%)	0 (0%)	1

Abbreviations: SD, standard deviation.

To observe the pathological characteristics of the surgical resection site, we analyzed H&E sections of the mucosal layer tissue that was peeled off by ESD in the FS hydrogel group. Figure 8C displays the mucosal tissue that was peeled off during the ESD procedures and its corresponding sections. A part of the FS hydrogel that was still in the solid state still adhered to the esophageal mucosa from which it was peeled off, and the edges of the electrosurgical knife cuts were visible. H&E staining was performed on the mucosal section that was cut. The complete mucosal section image, which is circled by black ellipses in the section image, showed no apparent damage to the cells. This suggests that FS prevents potential mucosal damage caused by normal saline and other SIM, which can cause the lesions to peel off during ESD and maintain their complete pathological morphology. This enables clinicians to make accurate pathologic staging diagnoses and design the most suitable treatment plan for the patient. The findings indicate that our FS hydrogel can offer durable and safe mucosal elevation in the GI tract of large animals, safeguarding against perforation and bleeding during endoscopic procedures, and has the potential to be an excellent SIM material.

## 4 Conclusion

In this study, we developed a temperature-sensitive hydrogel material that is simple to synthesize, cost-effective, safe, easy to store, and able to assist ESD in overcoming the clinical problem. FS hydrogel exhibits controlled gelation, injectability, and a stable structure. Meanwhile, numerous experiments have demonstrated that FS hydrogels have good biocompatibility. Most importantly, the FS hydrogel can provide long-lasting mucosal elevation as SIM for *in vitro* ESD compared with normal saline, and the stripped lesion tissue retains intact pathologic morphology. Based on these properties, the FS hydrogel may be as an ideal SIM for a wide range of endoscopic resection techniques, such as intestinal polypectomy or anti-esophageal stricture procedures, contributing to better therapeutic outcomes in endoscopic procedures.

## Data Availability

The raw data supporting the conclusion of this article will be made available by the authors, without undue reservation.
