# Supplementary figures and images for: Injectable temperature-sensitive hydrogel facilitating endoscopic submucosal dissection
Source: Front Bioeng Biotechnol. 2024 Apr 29;12:1395731. doi: 10.3389/fbioe.2024.1395731 (PMC11089129; doi:10.3389/fbioe.2024.1395731)

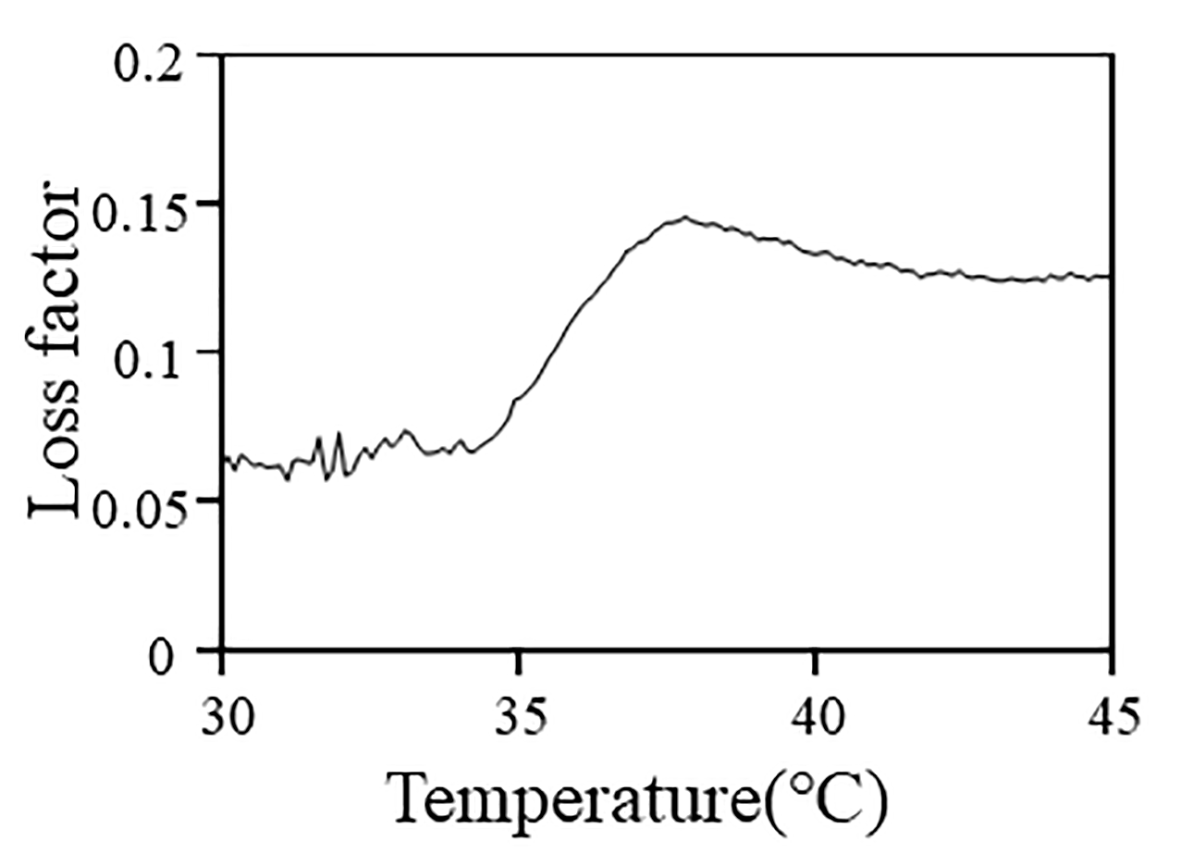

Supplement: Supplementary file 1 [file Image1.TIF]
