# Supplementary material for: Injectable temperature-sensitive hydrogel facilitating endoscopic submucosal dissection
Source: Front Bioeng Biotechnol. 2024 Apr 29;12:1395731. doi: 10.3389/fbioe.2024.1395731 (PMC11089129; doi:10.3389/fbioe.2024.1395731)
Supplement: Supplementary file 4 [file DataSheet1.docx]

Supplementary Material

Injectable Temperature-Sensitive Hydrogel Facilitating Endoscopic Submucosal Dissection.

Ruifen Xu1†, Xiaoyu Yang2†, Tong Yi2, Tao Tan3.5, Zhongqi Li5, Xuyang Feng4, Jing Rao1, Pinghong Zhou5, Hao Hu5*, Yonghua Zhan2*

*** Correspondence:** Hao Hu: hu.hao1@zs-hospital.sh.cn; Yonghua Zhan: yhzhan@xidian.edu.cn

# Supplementary Figures and Tables

#
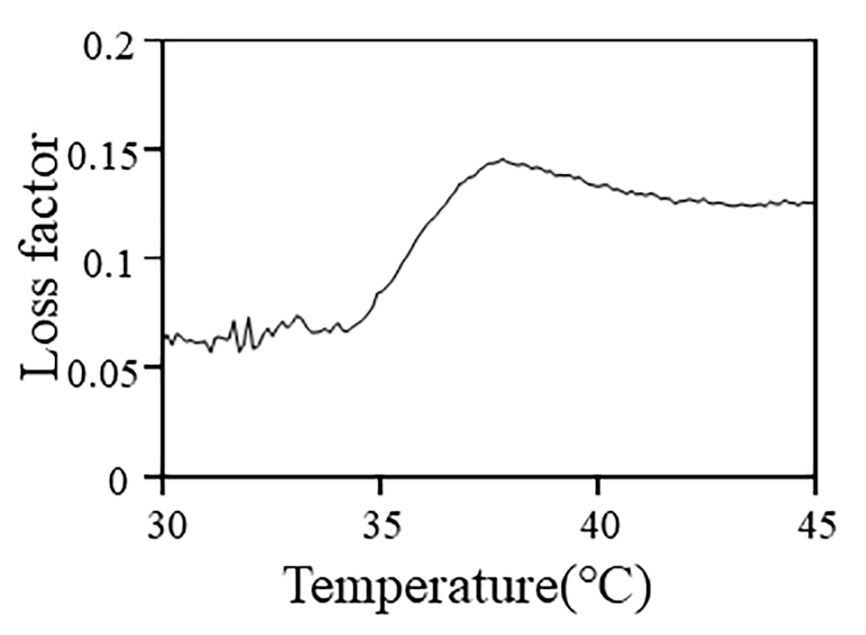


**Supplementary Figure 1.** The loss factor of the FS hydrogel.

**Supplementary Table 1.** FS Hydrogel Formulation Exploration.

| F127/g | sodium alginate/g | ultrapure water/mL | transition temperature /°C |
| --- | --- | --- | --- |
| 2 | 0.02 | 10 | 32 |
| 2 | 0.01 | 10 | 32 |
| 1.95 | 0.02 | 10 | 33 |
| 1.95 | 0.01 | 10 | 34 |
| 1.9 | 0.02 | 10 | 34 |
| 1.9 | 0.01 | 10 | 35 |
| 1.9 | 0.005 | 10 | 35 |
| 1.88 | 0.02 | 10 | 34 |
| 1.85 | 0.02 | 10 | 34 |
| 1.85 | 0.01 | 10 | 34 |
| 1.85 | 0.005 | 10 | 35 |
| 1.85 | 0.005 | 10 | 35 |
| 1.82 | 0.02 | 10 | 35 |
| 1.82 | 0.01 | 10 | 36 |
| 1.82 | 0.005 | 10 | 36 |
| 1.8 | 0.01 | 10 | 37 |
